# Synergistic Effect on the Thermomechanical and Electrical Properties of Epoxy Composites with the Enhancement of Carbon Nanotubes and Graphene Nano Platelets

**DOI:** 10.3390/ma12020255

**Published:** 2019-01-14

**Authors:** Yi-Ming Jen, Jui-Cheng Huang

**Affiliations:** Department of Mechanical and Mechatronic Engineering, National Taiwan Ocean University, Keelung 20224, Taiwan; 10272036@ntou.edu.tw

**Keywords:** nanocomposites, synergistic effect, graphene nanoplatelet (GNP), multi-walled carbon nanotube (MWCNT), thermomechanical, sheet resistance

## Abstract

The synergetic effect of adding multi-walled carbon nanotubes (MWCNTs) and graphene nanoplatelets (GNPs) on the thermomechanical properties and electric resistance of epoxy polymers were experimentally analyzed in this study. The total content of two employed carbon fillers were kept constant at 0.4 wt %, and seven filler ratios between two fillers (MWCNTs:GNPs), i.e., 10:0, 1:9, 3:7, 5:5, 7:3, 9:1, and 0:10, were considered in the experimental program to investigate the influences of employed nano-filler ratios on the viscoelastic and electrical properties of the studied nanocomposites. The thermomechanical properties and the sheet resistance of the nanocomposites were analyzed using a dynamic mechanical analyzer and four-point probe, respectively. Moreover, the thermogravimetric analyzer was utilized to measure the pyrolysis temperature of the nanocomposites. Experimental results show that the synergistic effect of adding two nano-fillers were clear for the improvement of the storage moduli, glass transition temperatures, and electric conductivity. Oppositely, the employment of two fillers has a slight effect on the pyrolysis temperatures of the studied nanocomposites. The composites with the MWCNT:GNP ratio of 1:9 display the most apparent enhancement of the thermomechanical properties. The improvement results from the uniform distribution and the high aspect ratio of GNPs. The addition of a small amount of MWCNTs provides more linkage in the matrix. Moreover, the specimens with the MWCNT:GNP ratio of 1:9 shows remarkable electrical properties, which result from the large contact surface areas of GNPs with each other. The employment of few MWCNTs plays an important bridging role between the layered GNPs.

## 1. Introduction

With the demand of electromagnetic shielding devices, conductive coatings, and antistatic materials, the conductive polymer materials have attracted more attention recently [1,2,3]. The electrical and thermal conductivities of polymers can be achieved by adding two or more carbon nano-fillers with different geometries in the matrix. Among three geometrically different types of carbon nano-fillers, i.e., particles, tubes, and platelets, the carbon nanotube and graphene are the most popular candidates as fillers used to enhance the electrical and thermal conductivities of the polymer materials. Compared with the carbon fillers with other geometrical characteristics, the one-dimensional shape of CNTs makes them potential fillers in the polymer materials to enhance the electrical and thermal conductivities because of their high aspect ratios. Recently, the application of graphene as the nano-fillers to reinforce the properties of polymer materials has rapidly increased because graphene has larger aspect ratio than CNTs, which makes it easy for electrical and thermal conduction [4,5,6]. However, the problem of agglomeration caused by the metallic bonds and Van der Waals interactions between the graphene sheets increases the difficulty of dispersion in the polymer matrix [7,8]. To overcome the shortcoming of agglomeration, some studies proposed that adding CNTs in the graphene-based polymer composites improves the dispersion of carbon fillers [7,9]. The obvious synergistic effect on improving the dispersion of hybrid nano-fillers makes the interest to investigate the properties of CNT/graphene or CNT/graphene nano-platelet (GNP) hybrid nanocomposites grows rapidly.

In the past 20 years, the electrical properties [8,10,11,12,13,14] and thermal conductivity [11,13,15,16,17] of the polymer composites with hybrid CNTs and GNPs have been studied. In general, the addition of CNTs and GNPs can improve the electrical and thermal conductivities by reducing the percolation thresholds substantially. Unlike the improvement behaviors in electrical and thermal properties, the mechanical properties can be enhanced by using a fewer amount of hybrid nano-fillers in the polymer matrix. Some studies have illustrated that, under specific CNT/GNP ratios, the mechanical properties of the nanocomposites can be improved significantly [7,8,10,12,16,18,19,20,21]. The synergistic effects of hybrid CNT/GNP fillers have been confirmed on the tensile modulus, tensile strength, flexural modulus, flexural strength, and fracture toughness of the polymer composites. However, the past experimental studies concerning the synergistic effect of CNT/GNP hybrid fillers on the mechanical properties of polymer materials are almost performed at room-temperature environments.

Since the characteristics of polymer materials are sensitive to the temperature, and the applications of carbon nanocomposites often encounter the thermal problems due to the electrical performance and surrounding environment, the knowledge of the synergistic effect on the thermomechanical properties is urgently needed. The main purpose of the study is to experimentally analyze the rheological properties of MWCNT/GNP hybrid epoxy composites, and to understand the synergistic effect of MWCNT/GNP hybrid fillers on the viscoelastic properties of the studied nanocomposites. The electrical properties and thermal stability of the MWCNT/GNP hybrid nanocomposites will be also investigated herein to evaluate the effect of adding studied hybrid fillers on the electrical properties and thermal stability. In the study, the filler ratios between two employed reinforcements will be an important variable to elucidate the influence of filler ratios on the previously mentioned properties of the nanocomposites.

## 2. Experimental Method

### 2.1. Materials and Specimen Preparation

**Figure 1:**
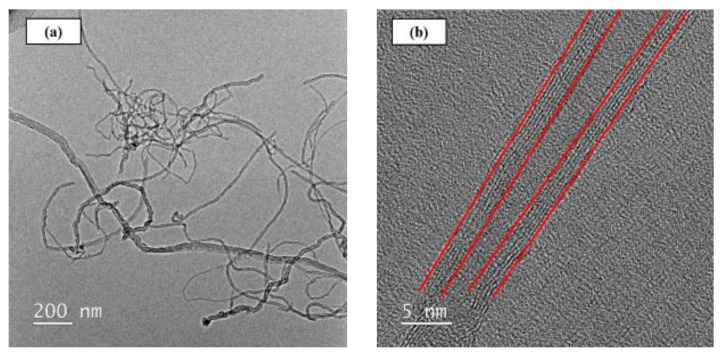
TEM images of as-received MWCNTs: (**a**) ×200, (**b**) ×10 k.

The matrix material employed in the present study was a two-part bisphenol A type epoxy (EPO-RT 90) supplied by Epotech Composite Corporation, Taichung, Taiwan. The mixing ratio between the epoxy resin and the hardener is 100:35. Applied Nanotechnologies, Inc., Chapel Hill, North Carolina, USA, provided the MWCNTs utilized. The purity of the studied MWCNTs is larger than 95%. Figure 1a,b shows the transmission electron microscopy (TEM, JEM-ARM200FTH, JEOL Ltd., Tokyo, Japan) images of the as-received MWCNTs taken at different magnifications. It is seen from Figure 1a that the length and the diameter of the employed MWCNT are approximately 2 μm and 10 nm, respectively. The magnified image of Figure 1b displays the six-layered wall structure of the as-received MWCNTs.

**Figure 2:**
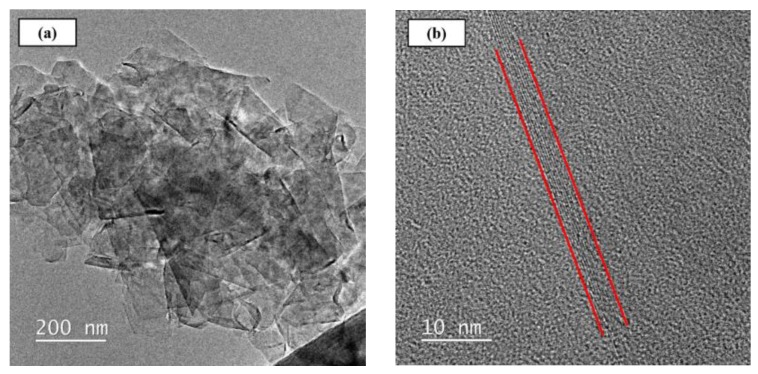
TEM images of as-received GNPs: (**a**) ×200, (**b**) ×10 k.

The GNPs with the designation of KNG-150 were supplied by Xiamen Knano Graphene Technology Corporation (Xiamen, China). The purity of the studied GNPs is larger than 99.5% and the specific surface area is around 40 to 60 m^2^/g. Figure 2 shows the TEM images of employed GNPs. Figure 2a shows that the average areal size of the as-received GNP is around 200 nm × 200 nm. The enlarged image as shown in Figure 2b displays that the GNP is characterized with the 10-layer sheet structure, and the lateral size of the studied GNPs is 3 nm.

Seven types of specimens with different filler weight ratios were prepared for future inspections. The total content of two employed fillers are controlled at 0.4 wt %. According to a preliminary study on the room-temperature mechanical properties of MWCNT/epoxy composites with various filler contents, the nanocomposites with 0.4 wt % MWCNT have the optimal static stiffness and strength compared with those with other MWCNT contents. In the present study, the total content of two nano-fillers of the studied specimens was controlled at 0.4 wt %, and the specimens with various MWCNT:GNP ratios were prepared by replacing different contents of MWCNTs with GNPs to investigate the synergistic effect of MWCNT/GNP hybrid fillers on the thermomechanical and electrical properties. The considered MWCNT:GNP ratios in the specimen preparation are 0:10, 10:0, 1:9, 3:7, 5:5, 7:3, and 9:1. The specimens with the filler ratios of 0:10 and 10:0 represent the epoxy matrix of the specimens, which were reinforced with GNPs and MWCNTs only, respectively. In addition, the specimens of neat epoxy were also prepared for the comparison purpose.

In preparing the nanocomposites specimens, the sodium dodecyl sulfate (SDS) was utilized as the surfactant to form beneficial groups on the surfaces of MWCNTs and GNPs. The SDS was added in acetone at 60 °C with mechanical stirring for 30 min. Then the nano-fillers were added in the previously mentioned solution with 10 min of mechanical stirring and 10 min of ultra-sonication to obtain the uniform distribution of the nano-fillers in the acetone solution. Next, the epoxy resin was added in the solution with 10 min mechanical stirring followed by 10 min ultra-sonication (LEO 3002, Yeong-Shin Co., Hsinchu, Taiwan). The solution was then stirred at 100 °C until the acetone is completely volatilized. The hardener was added in the solution with ultra-sonication and low-speed mechanical stirring for 30 min. The viscous solution was vacuumed for 1 h to remove the bubbles. The de-gassed solution was then poured into a mold and vacuumed again at 100 °C for 1 h. At last, the resin was cured at 1 atm and 100 °C for 8 h to obtain the solidified nanocomposites.

### 2.2. Experiments

In the study, the effectiveness of treatment of carbon nano-fillers using SDS was inspected using a Fourier transform infrared spectroscopy (FT-IR, FT/IR 4200, Jasco Inc., Easton, PA, USA). The morphological study was performed on the nanocomposites using a scanning electron microscope (SEM, JSM-6330F, JEOL Ltd., Tokyo, Japan) to examine the degree of nano-filler dispersion in the epoxy matrix. To study the thermomechanical properties of the fabricated nanocomposites with various filler rations, the bending-mode dynamic mechanical analyzer (DMA, Q800, TA Instruments Inc., New Castle, DE, USA) was employed to evaluate the viscous constants and rheological properties. The glass transition temperatures of the specimens *T_g_* were also obtained by using the DMA inspection. The loading frequency of the DMA tests was 3 Hz, and the measured temperature ranged from 15 to 145 °C. For each type of studied composite with specific filler ratio, three samples were prepared and tested for DMA tests to examine the repeatability of obtained results.

To study the electrical properties of the nanocomposites, an Everbeing EB-6 analytical probe station (Hsinchu, Taiwan) was used to measure the sheet resistances of the prospered specimens by using the four-point probe technique with a 2 mm pin interval. The studied nanocomposites with low hybrid filler concentrations (0.4 wt %) are expected to have the electrical conductivity near or below the percolation threshold of MWCNT/GNP/epoxy composites and the quantum tunneling effect is significant in the electrical conductivity [9,22,23,24,25]. The reason is that, according to the authors’ previous experimental results, under the same filler/matrix materials, filler concentrations, and specimen preparation conditions, the values of the percolation thresholds for the MWCNT/epoxy and GNP/epoxy composites are 3.5 and 0.9 wt %, respectively. The voltage of 50 V was supplied by the high voltage source in the resistance measurement of the neat epoxy specimens because of the insulated property of the polymer materials. The voltage applied for the sheet resistance measurement on the studied specimens with hybrid nano-fillers was controlled constant at 5 V to avoid the incorrect readings in the four-point probe measurements due to the quantum tunneling effect. The measuring capacity of the amperometer ranged from 10 pA to 1.05 A. Each sample with a specific filler ratio was measured seven times to obtain the average value for the resistance and ensure the results were reproducible. Furthermore, a thermogravimetric analyzer (TGA, Ssc5000 TG/DTA 300, Seiko Instruments Inc., Chiba, Japan) was adopted to assess the thermal stability, and the pyrolysis temperatures of the studied nanocomposites *T_d_* were also obtained. The TGA tests were performed in nitrogen. The measured temperature of the TGA inspection ranged from 25 to 800 °C with the ramp rate of 10 °C/min. The TGA tests for the studied nanocomposite specimens with identical filler ratio were repeated three times to ensure the reliability of obtained data.

## 3. Results and Discussion

**Figure 3:**
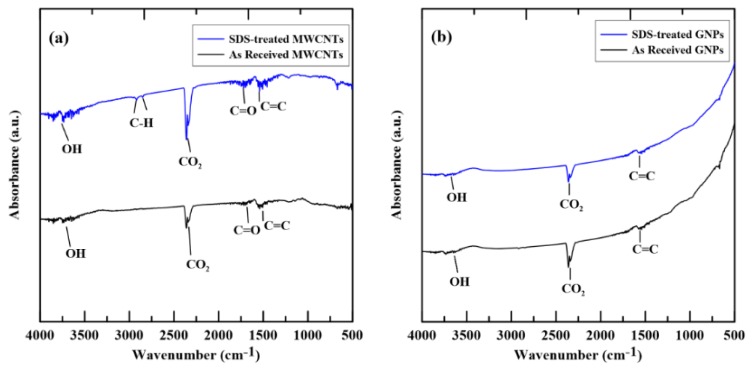
Comparison of the FT-IR spectra between the as-received and SDS treated carbon nano-fillers: (**a**) MWCNTs and (**b**) GNPs.

Figure 3a,b shows the FT-IR spectra of the employed MWCNTs and GNPs, respectively. In each figure, the spectra of as-received and SDS-treated fillers are depicted together to examine the results of treatment. As shown in Figure 3a, the –OH, –C=O, and –C=C peaks were observed both for the as-received and SDS-treated MWCNT spectra. The peaks mentioned above for the SDS treated MWCNT spectrum shows slightly stronger than that of as-received MWCNT, and tiny C–H peaks are found in the SDS treated MWCNT spectrum. Figure 3b shows only –OH and –C=O peaks presented and no obvious difference was found between the spectra of the SDS treated and as-received GNPs. The results shown in Figure 3a,b indicates that the SDS treatment has negligible effects on the fabrication of beneficial functional groups on the surfaces of nano-fillers for the formation of crosslink with the polymer matrix.

**Figure 4:**
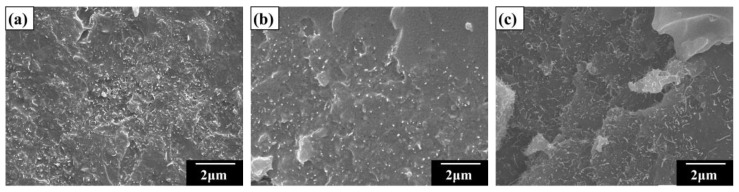
SEM images of the epoxy composites with the MWCNT:GNP ratios of (**a**) 10:0, (**b**) 5:5, and (**c**) 7:3 obtained after the SDS treatments.

Figure 4a–c shows some SEM image examples to elucidate the effect of SDS treatment on filler dispersion in the epoxy matrix. These examples are taken from the studied epoxy composites with the MWCNT:GNP ratios of 10:0, 5:5, and 7:3. From the morphological studies of these figures, the uniform dispersion of employed nano-fillers was observed after the SDS treatment. This indicates that the employment of surfactant improves the degree of dispersion significantly. Since the structure of the surfactant SDS has both the hydrophilic and hydrophobic ends, it is often used to solve the agglomeration problem of the carbon nano-fillers. The hydrophobic end of the SDS can adhere to the carbon filler, while the hydrophilic end improves the dispersion of the carbon fillers in the polymer matrix [26,27].

**Figure 5:**
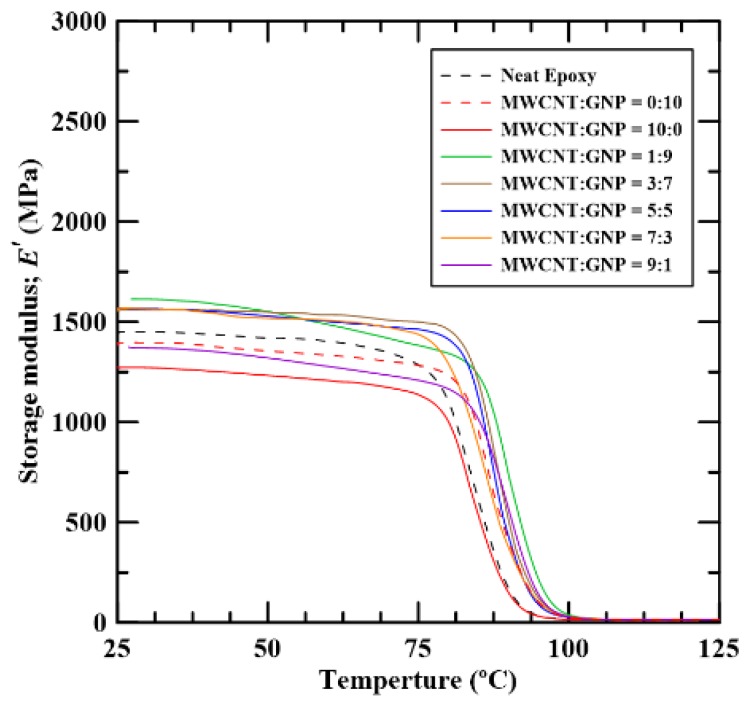
Temperature dependence of the storage modulus *E′* of the epoxy composites with various MWCNT:GNP ratios.

**Figure 6:**
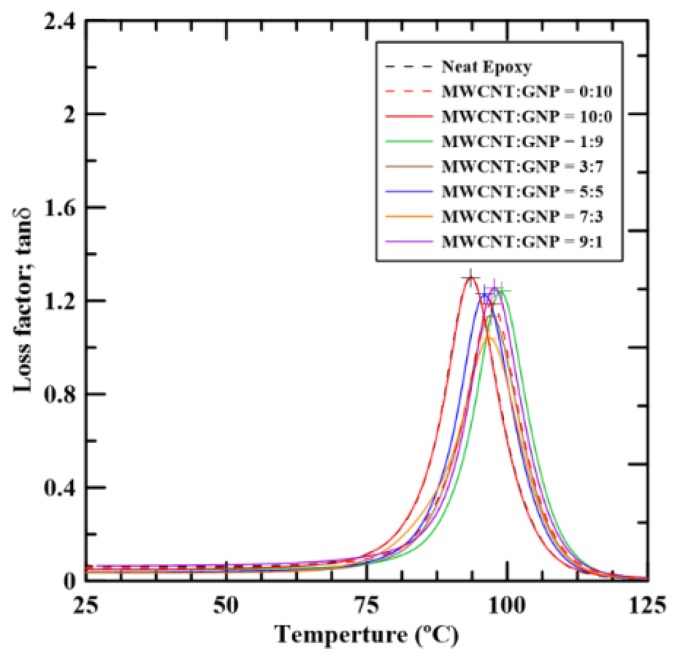
Temperature dependence of the loss factor tan δ of the epoxy composites with various MWCNT:GNP ratios.

Figure 5 and Figure 6 show the thermomechanical results experimentally obtained using DMA. It is noted that the curves shown in Figure 5 and Figure 6 obtained from three repeated tests for each type of specimen almost converge together, which indicates that the repeatability of the DMA test is verified. The curves shown in Figure 5 and Figure 6 select the ones where the obtained glass transition temperature is closest to the average value among the three identical DMA tests for the same type of specimens. Figure 5 depicts the temperature dependence of storage modulus *E′* of the studied nanocomposites with different MWNT/GNT ratios. It shows that, in the glassy region, the composites with single carbon nano-filler decrease the storage moduli of the neat epoxy. However, the employment of hybrid fillers in the epoxy improves the storage moduli significantly except for the specimens with the MWCNT:GNP ratio of 9:1. The hybrid composites with the MWCNT:GNP ratio of 1:9 display the best synergistic effect on the thermomechanical properties for the corresponding values of the storage moduli, which are higher than those with other filler ratios. The improvement results from the uniform distribution and the high aspect ratio of MWCNTs. The addition of a small amount of GNPs provides more linkage in the matrix. Surveying past literature reveals that the synergistic effect of the MWCNT/GNP hybrid fillers on the thermomechanical properties of epoxy composites is scarcely studied. The optimal filler ratios obtained by a few investigations on the room-temperature mechanical properties of the MWCNT/GNP/epoxy composites are diverse [7,10,16,21]. Among these studies, the drastic (1:9 or 9:1) and equal (5:5) ratios are frequently reported. Yang et al. [16] found the epoxy composites with the MWCNT:GNP ratio of 1:9, which have higher tensile modulus and strength than those with other filler ratios. The greater contact surface between two fillers under the specific ratio is considered the main mechanism of reinforcement.

**Figure 7:**
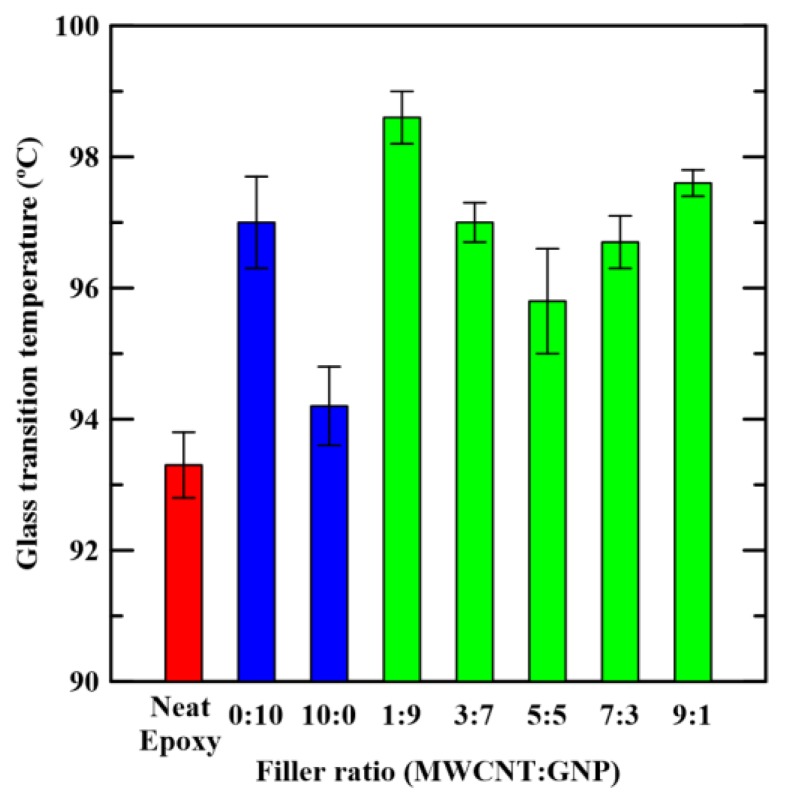
Variation of measured glass transition temperatures of the studied nanocomposites *T_g_* with the employed filler ratios in the specimen preparation.

Figure 6 shows the temperature-dependent loss factor tan *δ* curves of the studied nanocomposites with various filler ratios. It shows that peaks of loss factor curves decrease with the addition of carbon nano-fillers in the pristine epoxy. Moreover, the loss factor curves tend to shift to the right when the epoxy specimens are added with the nano-fillers. The trend to the left of the loss factor curves for the hybrid nanocomposites with appropriate filler ratios is stronger than those with single filler. In Table 1, it is reported, for all samples analyzed, the glass transition temperature, *T_g_* obtained from the temperature of maximum peak of Figure 6. As shown in Figure 6, these peaks are marked by the cross symbols. Figure 7 shows obtained glass transition temperatures of the studied nanocomposites. The figure shows that glass transition temperatures of the nanocomposites with single carbon nano-filler are higher than that of the neat epoxy. Moreover, the hybrid nanocomposites with appropriate filler ratios can further increase the glass transition temperatures. The studied composites with the MWCNT:GNP ratio of 1:9 have the highest glass transition temperatures, which implies that the most significant synergistic effect on the thermomechanical properties of GNP-epoxy composites can be obtained by replacing a small amount of nano-fillers with MWCNTs. The network constituted by two different types of carbon nano-fillers with high stiffness and strength can expand the temperature region of glassy states of the studied nanocomposites effectively.

**Figure 8:**
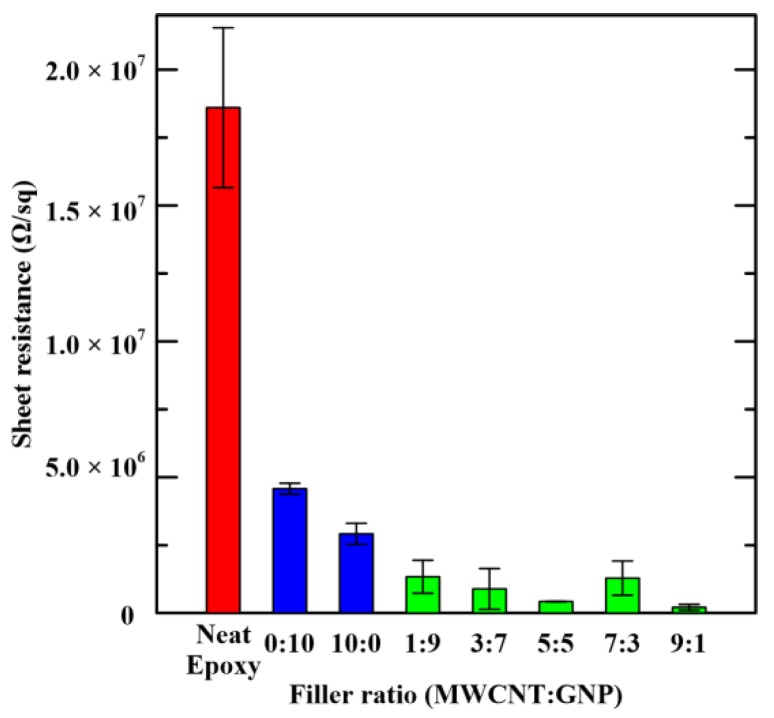
Variation of measured sheet resistances of the studied nanocomposites with the employed filler ratios in the specimen preparation.

Table 1 lists the measured sheet resistance of the studied nanocomposites. The results are also depicted in Figure 8. The data of pristine epoxy are also shown in the figure for comparison. It is evident that adding single carbon nano-filler can reduce the electrical resistance significantly. The values of obtained sheet resistance for the MWCNT-epoxy and GNP-epoxy composites were six and four times lower than that of the neat epoxy, respectively. Furthermore, the employment of hybrid fillers in the epoxy matrix future lowers the sheet resistance when compared with that of single filler composites. The resistance of the pristine epoxy decreased several times when the hybrid fillers were added in the matrix. The network composed by two geometrically different carbon nano-fillers effectively enhances the conductive properties. Among the studied composites with two types of fillers, the composites with the MWCNT:GNP ratio of 9:1 displayed the lowest resistance. The sheet-like structure of GNPs can increase the contact area between each other while the electrical conductivity of the studied nanocomposites is increased. The addition of a small amount of MWCNTs can constitute bridges between GNPs, and can prevent the agglomeration of GNPs to enhance the dispersibility of GNPs. The optimal MWCNT:GNP filler ratio for the electrical conductivity of epoxy composites was seldom studied before. The MWCNT:GNP ratios employed in the specimen preparation of References [10,13] were controlled at 1:1. He et al. [13] prepared the MWCNT/GNP/epoxy composite specimens with different filler ratio to study the synergistic effect of the hybrid fillers on the electrical conductivity. The composites with more GNPs were found to have higher conductivity. However, the high loading of 10 wt % was designated for the total content of two fillers in the specimen fabrication.

**Figure 9:**
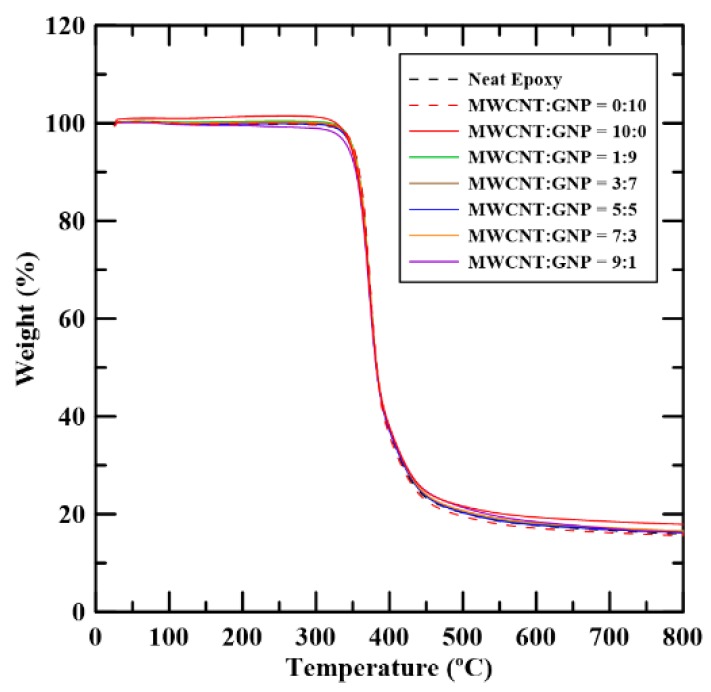
Relationship between the weight loss of the epoxy composites with various MWCNT:GNP ratios and temperatures obtained using TGA analysis.

**Figure 10:**
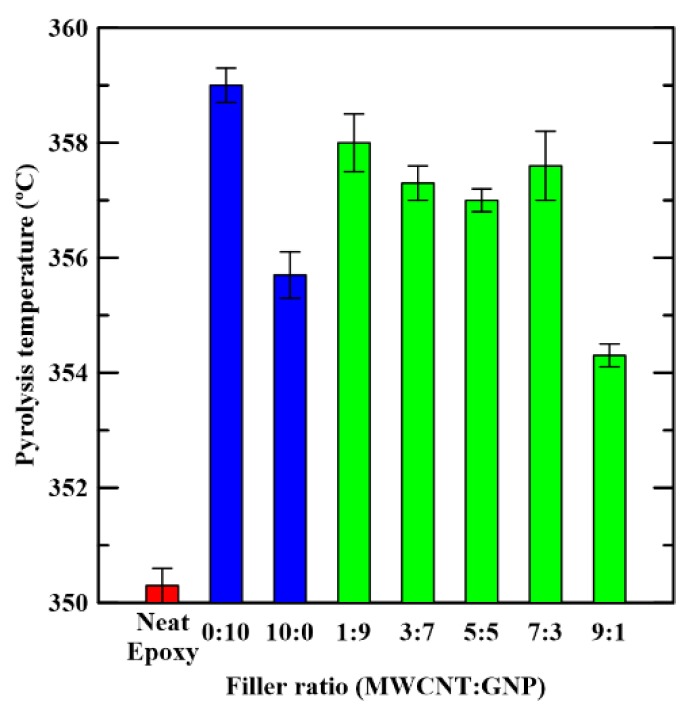
Variation of measured pyrolysis temperatures of the studied nanocomposites *T_d_* with the employed filler ratios in the specimen preparation.

Figure 9 shows the weight loss curves of the studied nanocomposites with different filler ratios obtained using TGA. The curve depicted here is the one where the corresponding pyrolysis temperature is closest to the average value obtained from the five identical TGA tests for the same type of specimens. It shows that these weight loss curves of the fabricated samples with various filler ratio are almost overlapping. This implies that the effects of filler ratios and the number of filler types are tiny on the thermogravimetric properties. The pyrolysis temperature of the studied sample *T_d_* was obtained from the curve since the temperature corresponded to 10% weight loss. The observed pyrolysis temperatures of the specimens with various filler ratios are listed in Table 1. Figure 10 shows the pyrolysis temperatures of the studied nanocomposites with various filler ratios. It is evident that adding carbon nano-fillers can increase the pyrolysis temperatures of the neat epoxy by 5 to 8 °C. However, the difference of the pyrolysis temperatures between the nanocomposites with single and hybrid filler systems is minor, which indicates that the synergistic effect of two types of carbon nano-fillers on the thermal decomposition properties is not noticeable.

## 4. Conclusions

The thermomechanical, electrical, and thermal stability properties of the epoxy composites reinforced by MWCNT and GNP hybrid fillers were experimentally studied herein to elucidate the synergistic effect of hybrid carbon nano-fillers on these properties of the studied composites. Some conclusions can be summarized as follows.

(1) The results of morphological study show that the uniform dispersion of employed nano-fillers in the epoxy matrix can be obtained by applying the SDS treatment. The special hydrophobic and hydrophilic ends of SDS can avoid the agglomeration of carbon fillers and improve the dispersion effectively. Moreover, the uniform dispersion of nano-fillers in the specimen preparation ensures the repeatability of tests.
(2) A strong synergistic effect of the hybrid carbon nano-fillers was found for the studied nanocomposites to improve the storage moduli and increase the glass transition temperatures.
(3) The high aspect ratio of GNPs and the additional linkage provided by the few MWCNTs contribute to the significant synergistic effect on the thermomechanical properties of the composites with the MWCNT:GNP ratio of 1:9.
(4) Comparing the studied composites with other filler ratios, the composites with the MWCNT:GNP ratio of 1:9 display a more intensive synergistic effect of the hybrid nano-fillers for the enhancement of electrical properties.
(5) The large contact areas between the GNPs and the bridging effect provided by the MWCNTs is the main synergistic mechanism of the hybrid nano-fillers on the electrical properties of the studied composites with the MWCNT:GNP ratio of 1:9.
(6) Although the addition of carbon nano-fillers can increase the pyrolysis temperatures of the studied composites, the synergistic effect of the hybrid fillers on the thermal stability of epoxy composites is not evident.

## Figures and Tables

**Table 1:** Measured values of glass transition temperatures, sheet resistances, and pyrolysis temperatures for the epoxy composites with various MWCNT:GNP ratios.

Filler Ratio (MWCNT:GNP)	Glass Transition Temperature *T_g_* (°C)	Sheet Resistance (×10^5^ Ω/sq)	Pyrolysis Temperature *T_d_* (°C)
Neat Epoxy	93.3 ± 0.5	186 ± 29.4	350.3 ± 0.4
0:10	97.0 ± 0.7	45.8 ± 2.02	359.0 ± 0.3
10:0	94.2 ± 0.6	29.2 ± 3.87	355.7 ± 0.4
1:9	98.6 ± 0.4	13.4 ± 6.09	358.0 ± 0.5
3:7	97.0 ± 0.3	8.92 ± 7.49	357.3 ± 0.3
5:5	95.8 ± 0.9	4.28 ± 0.13	357.0 ± 0.2
7:3	96.7 ± 0.4	12.9 ± 6.28	357.6 ± 0.6
9:1	97.6 ± 0.2	2.15 ± 1.11	354.3 ± 0.2
